# Puffy hand syndrome with histopathological evidence of a cutaneous granulomatous reaction to starch in the setting of prior intravenous drug use

**DOI:** 10.1016/j.jdcr.2023.07.034

**Published:** 2023-08-09

**Authors:** Tatiana M. Barrera, Omar Venegas, Tiaranesha Jackson, Yacine Sow, Christopher I. Wachuku, Rosalie Elenitsas, Susan Taylor, Nicholas Mollanazar

**Affiliations:** a University of California, Riverside School of Medicine, Riverside, California; b University of Illinois College of Medicine, Peoria, Illinois; c Morehouse School of Medicine, Atlanta, Georgia; d Rutgers, Robert Wood Johnson Medical School, New Brunswick, New Jersey; e Department of Dermatology, Perelman School of Medicine, University of Pennsylvania, Philadelphia, Pennsylvania

**Keywords:** buprenorphine, cutaneous granuloma, foreign body granuloma, heroin, intravenous drug use, IVDU, maltese cross, pregnancy, puffy hand syndrome, skin biopsy, starch

## Introduction

In the United States, 9.5 million people struggle with opioid misuse, of which 2.7 million meet the Diagnostic and Statistical Manual of Mental Disorders, Fifth Edition (DSM-5) criteria for opioid use disorder.^1^ Intravenous drug use (IVDU) can lead to cutaneous reactions associated with injected drug contaminants or fillers, including sugars, talc, and starch.^2^ Puffy hand syndrome (PHS)—a phenomenon associated with chronic IVDU—clinically presents as intermittent, painless, erythematous, nonpitting edema of the hands and upper extremities. Despite its nomenclature, PHS may involve the lower extremities, particularly in patients with a history of injections to their legs or feet.^3^ Though its pathophysiology is not entirely understood, it has been defined as a diagnosis of exclusion, with nonspecific histopathology and laboratory findings.^4^ We report a case of a pregnant patient with a multiple-year history of IVDU who presented with acquired lymphedema suggestive of PHS, likely secondary to a granulomatous reaction to starch. This suggests that specific histopathological findings may be associated with PHS, which challenges the nonspecific histopathological criteria associated with this diagnosis.

## Case report

A 34-year-old patient presented to dermatology at 28 weeks gestation following Obstetrics and Gynecology referral. The patient has a more than 2-year history of intermittent, disfiguring edema, erythema, and pruritus of the bilateral hands and lower legs. At the time of presentation, the patient was undergoing opioid dependence treatment with oral buprenorphine. Past medical history included: years of heroin self-injections to the hands and lower legs; positive hepatitis C virus antibody (RNA negative); bilateral hand cellulitis and tenosynovitis; and nonhealing, open leg wounds.

**Fig 1:**
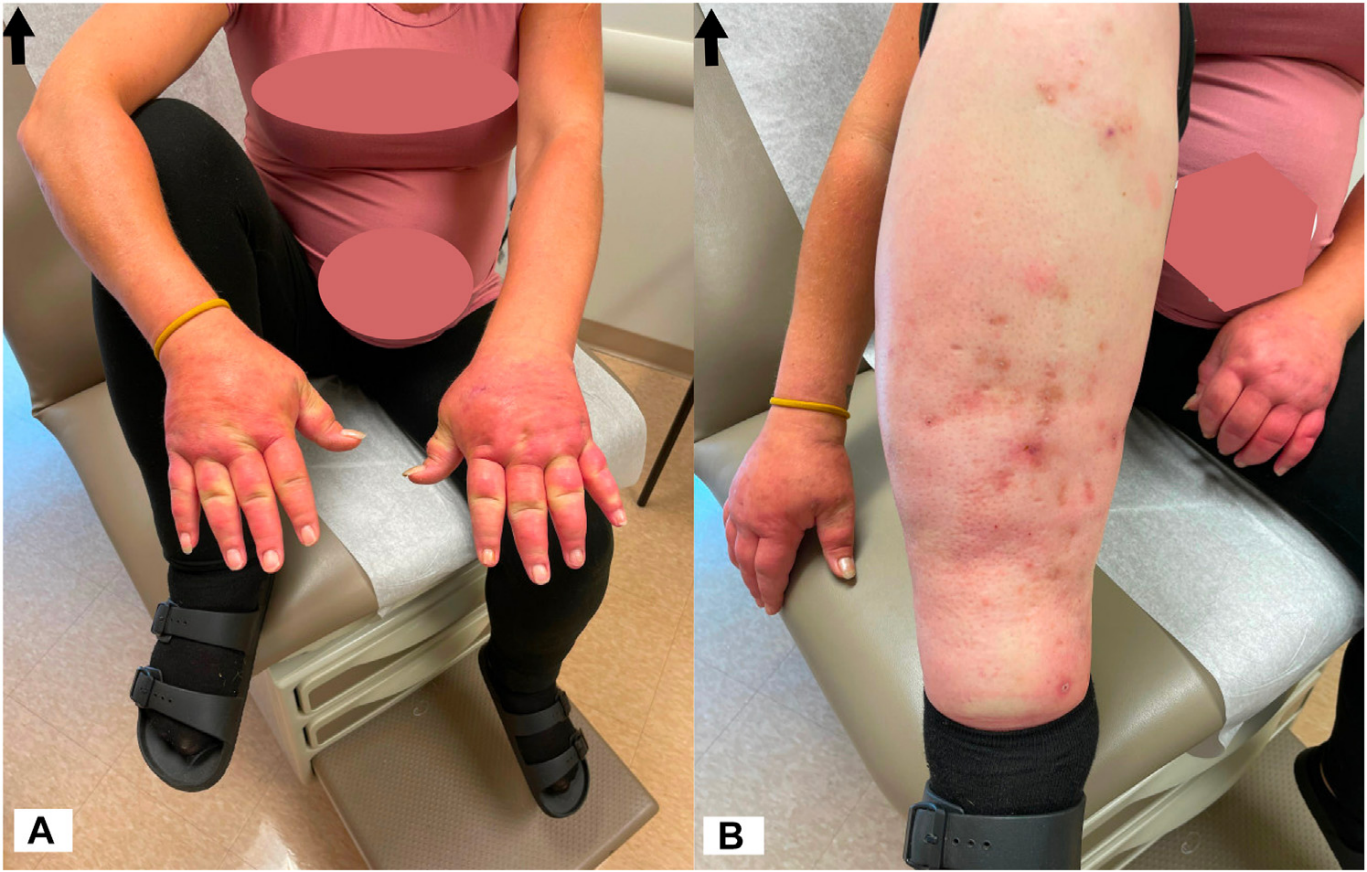
Clinical findings of puffy hand syndrome. Severe pitting, woody edema of all 4 limbs, (**A**), with nodular erythema of the dorsal hands, and (**B**), erythema underlying pitted, hyperpigmented scars of the *lower* limbs.

Physical examination identified severe pitting and woody edema of all four limbs, with nodular erythema of the dorsal hands up to the wrists sparing metacarpophalangeal/proximal interphalangeal joint joints (Fig 1, *A*) and erythema underlying pitted, hyperpigmented scars of the lower legs (Fig 1, *B*).

Our differential diagnoses included lymphedema, angioedema, contact dermatitis, and mixed connective tissue disorder (MCTD). Though hand edema may be observed in MCTD, extreme edema of all four limbs is atypical. Thus, we performed serology and punch biopsies.

**Fig 2:**
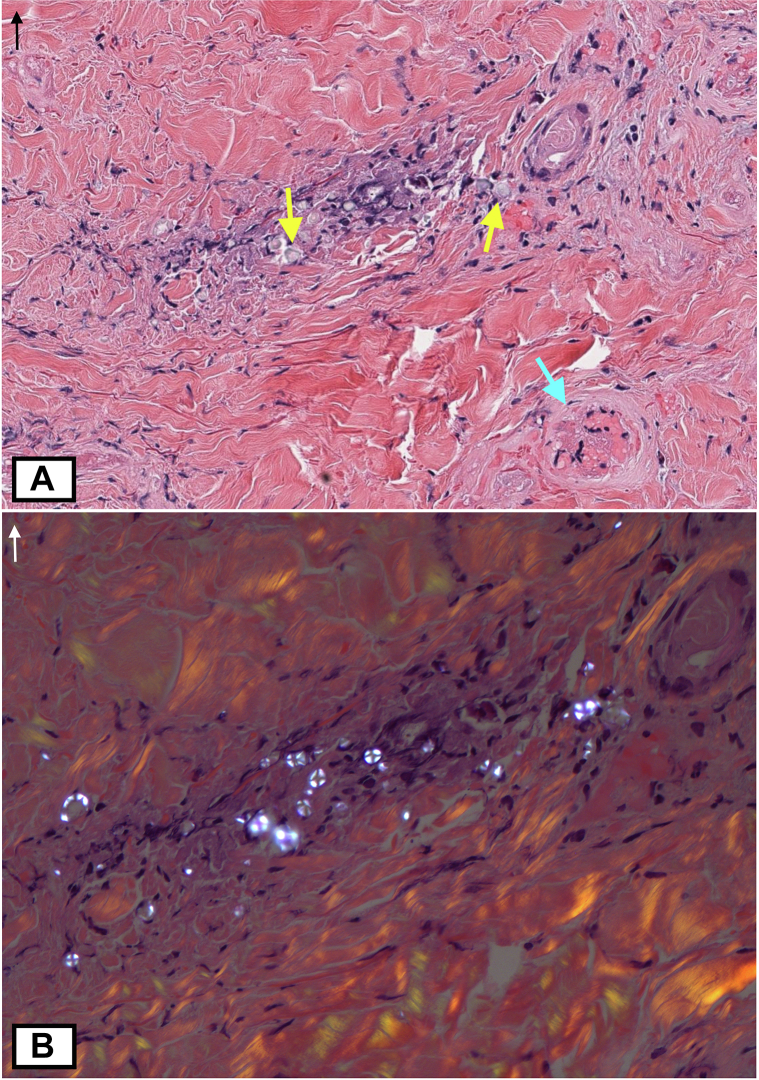
Histopathological findings of puffy hand syndrome. **A,** The *left* dorsal hand biopsy revealed perivascular inflammation small vessel engorgement (*blue arrows*), dermal necrosis, and foreign material (*yellow arrows*); (hematoxylin & eosin, ×350). **B,** Polariscopic examination shows foreign material in a Maltese cross pattern; (hematoxylin & eosin with polaroscopy, ×400).

Punch biopsies of the left dorsal hand demonstrated perivascular lymphocytic and neutrophilic infiltrate, small vessel engorgement, and dermal necrosis (Fig 2, *A*). The left hand and left lower limb biopsies revealed small foci of granulomatous inflammation with polarizable, refractile foreign material in a Maltese-cross pattern highly suggestive of starch (Fig 2, *B*).^2^^,^^5^

Serologies were positive for low creatinine kinase (24 U/L), elevated noncardiac c-reactive protein (1.30 mg/dL), and Immunoglobulin M anti-beta-2-glycoprotein (19 units). Lupus anticoagulant assays, anticardiolipin antibodies, and anti-Smith/antinuclear ribonucleoprotein antibodies (a hallmark of MCTD) were negative.

The histopathology was consistent with a cutaneous foreign body granuloma containing starch. The patient was referred to wound care for secondary skin infection and symptomatically treated with lymphedema massages. Although elevated immunoglobulin M anti-beta-2-glycoprotein can present in pregnancy, we referred the patient to hematology and rheumatology for antiphospholipid syndrome workup and management. Unfortunately, the patient was lost to follow-up.

## Discussion

Biopsies revealing pulmonary and retinal granulomas containing starch have been reported in patients with confirmed IVDU.^5^, ^6^, ^7^ Although some of these patients also presented with cutaneous findings, they did not undergo skin biopsies.^6^^,^^7^ This patient’s presentation is suggestive of PHS with histopathological evidence of a cutaneous, foreign body granuloma containing starch—a unique finding given that most skin biopsies in cases of PHS have not revealed specific histopathology.^4^^,^^8^ We postulate that starch was introduced to the limbs via injections and that the granulomatous reaction may help explain the pathogenesis of PHS.

Drugs like heroin may be adulterated with diluents of pharmacologically inactive substances (eg, mannitol, sucrose, starch) to increase quantity and profits.^2^^,^^5^ Starch is also an insoluble excipient that serves as a binder and disintegrant of oral medications, including stimulants and opiates (Table I).^2^^,^^5^ When heroin or dissolved tablets are not properly filtered before intravenous administration, particles of poorly soluble contaminants or excipients can be injected into veins and subcutaneous tissues, potentially resulting in fibrosis, necrosis, and granulomas.^2^

**Table I:** Substances commonly found as excipients of prescription opiate tablets^2^^,^^5^

Excipients	Prescription opiate tablets				Microscopic presentation		
	Morphine	Oxycodone hydrochloride	Buprenorphine	Buprenorphine/naloxone	Size	Shape	Polarization
Cellulose	X				20-200 μm	Matchstick/rod	Positive
Starch			X	X	8-12 μm	Round, polyhedral	Positive with maltese cross pattern
Talc	X	X			5-15 μm	Stacked plates, needle-like	Positive

Adapted from: McLean et al^2^ and Arboe and Tomashefski.^5^

The presence of intermittent, bilateral upper, and lower edematous extremities in our patient with past IVDU is suggestive of PHS, which can present years after the patient’s last injection.^8^^,^^9^ Proposed pathophysiology of PHS includes cutaneous, lymphatic, and infectious etiologies.^4^ Others have postulated that a weakened lymphatic system during pregnancy may play a role in accelerating the onset of PHS.^10^ Our pregnant patient’s skin biopsy supports the theory that repeated injections of insoluble substances contribute to local occlusion of lymphatics and fibrosis of surrounding tissues, leading to the development of PHS.^8^, ^9^, ^10^ Moreover, it underscores that positive histopathologic findings of foreign bodies specific to IVDU (Table I) should not discount the diagnosis. As exemplified by this case, patients with multi-limb edema or a history of injections to more than one limb may require several skin biopsies. However, inconclusive histopathological findings are common in PHS regardless of the number of specimens sampled. Clinicians and patients should therefore consider that skin biopsies may support the diagnosis but may not always identify the etiology.

Whichever the histopathological outcome, the prognosis and treatment approach for PHS are challenging. Although some cases in the literature have reported improvement with limb elevation and compression, others have resolved on their own.^3^^,^^8^ Notably, though initially intermittent, limb edema associated with PHS can become a chronic and even permanent condition for patients if left untreated.^8^^,^^9^ The disfigurement and limited treatment options associated with this condition can contribute to the psychosocial barriers of drug rehabilitation.^3^

PHS has been reported in up to 16% of patients with IVDU.^4^ Therefore, dermatologists should maintain clinical suspicion of PHS when encountering patients with edematous extremities, particularly in patients with a known history of IVDU. When considering PHS as a differential, a detailed social history should inquire about past and current IVDU (eg, substance type, duration of use, injection location, and use of a tourniquet).^3^^,^^9^ Furthermore, skin biopsies can identify underlying etiologies of PHS. Positive histopathological findings can serve as confirmatory support to dermatologists counseling patients on the associated long-term effects of PHS. A prompt diagnosis may guide therapies to mitigate chronic cutaneous and lymphatic injury. In addition, it can serve as an opportunity for physician-patient dialog on psychosocial concerns, referral to mental health services, and the prognosis of PHS.

## Conflicts of interest

None disclosed.
